# The prognostic value of arginase-1 and glypican-3 expression levels in patients after surgical intrahepatic cholangiocarcinoma resection

**DOI:** 10.1186/s12957-021-02426-9

**Published:** 2021-10-29

**Authors:** Zeyuan Qiang, Haofeng Zhang, Shuai Jin, Cao Yan, Zhen Li, Lianyuan Tao, Haibo Yu

**Affiliations:** 1 Department of Hepatobiliary Surgery, Henan University People’s Hospital, Henan Provincial People’s Hospital, Zhengzhou, 450003 China; 2 Department of Hepatobiliary Surgery, Medical College of Zhengzhou University, Zhengzhou, China; 3 Department of Pathology, Henan Provincial People’s Hospital, Zhengzhou, China

**Keywords:** Arginase-1, Glypican-3, Prognosis, Intrahepatic cholangiocarcinoma, Immunohistochemistry

## Abstract

**Background:**

The aim of this study was to investigate the prognostic value of arginase-1 (Arg-1) and glypican-3 (GPC-3) in patients with intrahepatic cholangiocarcinoma (ICC).

**Methods:**

Two hundred and thirty-seven patients with ICC were included in this study. All patients had undergone radical surgery and had complete clinical information. Immunohistochemistry was used to assess the levels of Arg-1 and GPC-3 in ICC tissues. Univariate and multivariate analyses were conducted to identify independent risk factors in ICC. The relationship between Arg-1 and GPC-3 levels and patient survival was determined using the Kaplan-Meier method.

**Results:**

High Arg-1 and GPC-3 expression levels were associated with poor prognosis in patients with ICC, and they could be as new prognostic biomarkers in ICC.

**Conclusion:**

Arg-1 and GPC-3 can serve as independent prognostic biomarkers in ICC.

**Supplementary Information:**

The online version contains supplementary material available at 10.1186/s12957-021-02426-9.

## Introduction

Intrahepatic cholangiocarcinoma (ICC) is an epithelial tumor originating from the secondary bile ducts of the liver. ICC is the second most common type of liver cancer and is an aggressive malignancy characterized by high rates of metastasis and poor prognosis [1]. Some studies have shown that hepatolithiasis, liver flukes, biliary duct cysts, hepatitis C infection, primary sclerosing cholangitis, and genetic polymorphisms are the major risk factors for ICC [2]. Most ICCs are diagnosed at an advanced stage, contributing to the poor prognosis of the disease [3]. The worldwide incidence of ICC is currently on the rise [4]. Surgical resection is currently the preferred treatment for ICC. After radical surgical resection of the tumor, the 5-year overall survival rate of patients with ICC is only 17–35% [5]. For patients who cannot be treated surgically, local treatment is often used clinically, including transcatheter hepatic arterial chemoembolization, percutaneous radiofrequency, and hepatic artery infusion, but they do not have a significant effect on survival [6, 7]. Hence, there is no effective way to evaluate and improve patient survival, the development of new methods to accurately predict relapse in high-risk patients is key to improving clinical outcome.

Arginase is an enzyme involved in the ornithine cycle in the liver, catalyzing the conversion of arginine to ornithine and urea. There are two arginase isoforms, Arg-1 and Arg-2 [8], which have the same biochemical effects but differ in tissue distribution and intracellular localization. Arg-1 is primarily found in the cytoplasm of hepatocytes [9]. Changes in Arg-1 expression may cause metabolic disorders and tumor development. Glypican-3 (GPC-3), a member of the glypican family, is anchored to the cell surface by glycosylphosphatidylinositol. The unique structure of GPC enables it to store and isolate cytokines, chemokines, and growth factors. It can both negatively and positively regulate cell growth depending on the cell type [10]. Mucin-1 (MUC1) is one of the main members of mucin family and is mainly expressed on the apical surface of glandular epithelial cells such as mammary gland, esophagus, lung, stomach, and pancreas. Its main role is to participate in the formation of physical barrier, lubrication, and protection of normal epithelial tissues and signal transduction [11].

Previous studies have shown that Arg-1, GPC-3, and MUC1 can promote the proliferation and metastasis of malignant tumors and serve as prognostic biomarkers in several solid tumors [12, 13]. However, there is still a lack of relevant research on Arg-1, GPC-3, and MUC1 in the clinical treatment of ICC. In the present study, we explored the expression levels of Arg-1, GPC-3, and MUC1 in ICC tissues. We also evaluated the relationship between the levels of Arg-1 and GPC-3 and the clinical features of ICC. This study paves the way for the development of markers to predict prognosis in patients with ICC.

## Materials and methods

### Patients

This study adhered to the principles of the Declaration of Helsinki and was approved by the research ethics committee of Henan University People’s Hospital. Informed consent was obtained from all study subjects. We followed 1798 patients treated with surgery for ICC between October 2009 and September 2019 at Henan Provincial People’s Hospital. The following inclusion criteria were used: (1) ICC diagnosis by pathology and imaging, (2) no adjuvant therapy before surgery, (3) no serious underlying conditions, (4) ICC treatment with radical surgery, (5) no history of other malignancies, and (6) availability of complete clinical data. Patients diagnosed with hepatocellular carcinoma or extrahepatic cholangiocarcinoma were excluded from the study. After excluding lost patients, the clinical data of a total of 237 patients were analyzed in this study.

### Follow-up

All patients were detected for tumor recurrence or metastasis by B-ultrasound, dynamic contrast-enhanced CT or dynamic contrast-enhanced magnetic resonance and blood biochemical examination. The patients were rechecked every 3 months within 3 years and every 6 months after 3 years. In case of tumor recurrence and metastasis, the patient’s physiological status, tumor size and location, and extrahepatic metastasis will be evaluated and then reoperation, radiotherapy, radiofrequency ablation, or other treatments will be performed.

### Immunohistochemistry

The expression levels of Arg-1 and GPC-3 in 237 resected ICC samples were evaluated by immunohistochemistry (IHC). All specimens were fixed in 4% neutral formaldehyde solution, dehydrated with gradient alcohol, embedded in paraffin, sectioned, and stained with hematoxylin-eosin (H&E). H&E-stained tissues were observed under a light microscope (Nikon, 80i, Japan). ICC tissue sections were also stained with the following antibodies: rabbit monoclonal anti-Arg-1 antibody (SP15; Jianlun Biology Technology, China), mouse monoclonal anti-GPC-3 antibody (ab129381; Abcam, USA), and rabbit monoclonal anti-MUC1 antibody (ab109185; Abcam, USA).

### IHC scoring

**Fig. 1:**
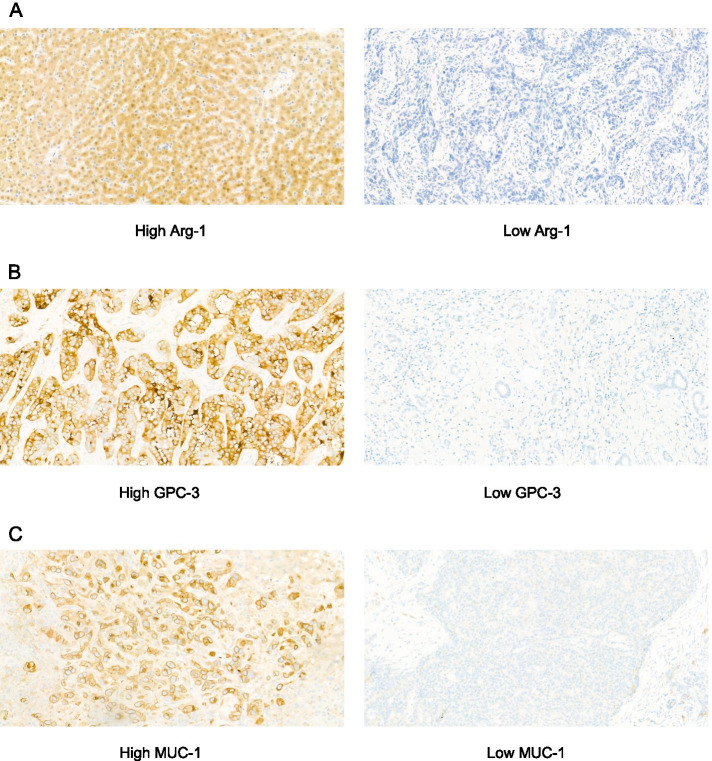
Immunohistochemistry staining for Arg-1, GPC-3, MUC1 in intrahepatic cholangiocarcinoma tumor tissues (400× magnification). **A** Arg-1 expression was divided into high (left panel) and low (right panel). **B** GPC-3 expression was divided into high (left panel) and low (right panel). **C** MUC1 expression was divided into high (left panel) and low (right panel)

IHC-stained tissues were evaluated by three pathologists blinded to the clinicopathological data. The fraction of positive cells was scored 0, 0% positive staining cells; 1, ≤ 25% positive cells; 2, 26–50% positive cells; 3, 51–75% positive cells; 4, ≥ 76% positive cells; then, the staining intensity was scored 0, negative; 1, weak; 2, moderate; 3, strong. The total IHC score was obtained by multiplying the staining intensity score by positively stained cell density score. Then, according to the total IHC score, we divided the samples into high expression group (the total IHC score ≥ 4) and low expression group (the total IHC score < 4) (Fig. 1) [14].

### Statistical analysis

All statistical analyses were conducted using SPSS 21.0. The association between the levels of Arg-1 and GPC-3 and clinicopathological features was assessed using the chi-square test. Survival analyses were conducted using the Kaplan-Meier method, and statistical significance was determined using the log-rank test. Univariate and multivariate analyses were used to assess the prognostic value of Arg-1 and GPC-3 expression levels. *P* values < 0.05 were considered statistically significant.

## Results

### Relationship between Arg-1 and GPC-3 levels and clinicopathological characteristics

The relationship between the levels of Arg-1 and GPC-3 and the clinicopathological characteristics of patients with ICC are explored in Table 1. High Arg-1 expression levels were associated with male (*P* = 0.043), HBV infection (*P* = 0.022), albumin levels over 35g/L (*P* = 0.018), and tumor size ≥ 5 cm (*P* = 0.047). High GPC-3 expression levels were associated with CA19-9 levels over 37 IU/L (*P* = 0.006) and tumor size ≥ 5 cm (*P* = 0.005; Table 1).

**Table 1:** Relationship between Arg-1 and GPC-3 levels and clinicopathological characteristics in patients with intrahepatic cholangiocarcinoma

Clinical parameter	Arg-1		*P* value	GPC-3		*P* value
	Low (%), *n* = 207	High, *n* = 30		Low (%), *n* = 201	High (%), *n* = 36	
Age (years)						
< 60	96 (46.4)	12 (40.0)	0.512	91 (45.3)	17 (47.2)	0.829
≥ 60	111 (53.6)	18 (60.0)		110 (54.7)	19 (52.8)	
Gender						
Female	103 (49.8)	9 (30.0)	*0.043	110 (54.7)	15 (41.7)	0.148
Male	104 (50.2)	21 (70.0)		91 (45.3)	21 (58.3)	
HBsAg						
Negative	145 (70.0)	27 (90.0)	^*^0.022	144 (71.6)	28 (77.8)	0.447
Positive	62 (32.4)	3 (10.0)		57 (28.4)	8 (22.2)	
AFP (ng/mL)						
< 25	140 (67.6)	25 (83.3)	0.081	140 (69.7)	25 (69.4)	0.980
≥ 25	67 (30.0)	5 (16.7)		61 (30.3)	11 (30.6)	
CEA (ng/mL)						
< 5	116 (56.0)	18 (60.0)	0.682	113 (56.2)	21 (58.3)	0.814
≥ 5	91 (44.0)	12 (40.0)		88 (43.8)	15 (41.7)	
CA19-9 (IU/L)						
< 37	128 (61.8)	13 (43.3)	0.054	127 (63.2)	14 (38.9)	^*^0.006
≥ 37	79 (38.2)	17 (56.7)		74 (36.8)	22 (61.1)	
TBIL (μmol/L)						
< 17.1	80 (38.6)	12 (40.0)	0.887	81 (40.3)	11 (30.6)	0.269
≥ 17.1	127 (61.4)	18 (60.0)		120 (59.7)	25 (69.4)	
ALB (g/L)						
< 35	148 (71.5)	15 (50.0)	0.018	142 (70.6)	21 (58.3)	0.142
≥ 35	59 (28.5)	15 (50.0)		59 (29.4)	15 (41.7)	
ALT (U/L)						
< 40	82 (39.6)	14 (46.7)	0.462	83 (41.3)	13 (36.1)	0.560
≥ 40	125 (60.4)	16 (53.3)		118 (58.7)	23 (63.9)	
Differentiation						
W+M	79 (38.2)	14 (46.7)	0.373	76 (37.8)	17 (47.2)	0.287
*P*	128 (61.8)	16 (53.3)		125 (62.2)	19 (52.8)	
Tumor size (cm)						
< 5	129 (62.3)	13 (43.3)	^*^0.047	128 (63.7)	14 (38.9)	^*^0.005
≥ 5	78 (37.7)	17 (56.7)		73 (36.3)	22 (61.1)	
Tumor number						
Single	143 (69.1)	24 (80.0)	0.221	142 (70.6)	25 (69.4)	0.884
Multiple	64 (30.9)	6 (20.0)		59 (29.4)	11 (30.6)	
Lymph node metastasis						
No	121 (58.5)	13 (43.3)	0.118	115 (57.2)	19 (52.8)	0.621
Yes	86 (41.5)	17 (56.7)		86 (43.3)	17 (47.2)	
Vascular invasion						
No	120 (58.0)	13 (43.3)	0.131	114 (56.7)	19 (52.8)	0.661
Yes	87 (42.0)	17 (56.7)		87 (31.0)	17 (47.2)	
Nerve invasion						
No	183 (88.4)	23 (76.7)	0.075	177 (88.1)	29 (80.6)	0.219
Yes	24 (11.6)	7 (23.3)		24 (11.9)	7 (19.4)	
Extrahepatic metastasis						
No	128 (61.8)	17 (56.7)	0.587	123 (61.2)	22 (61.1)	0.992
Yes	79 (38.2)	13 (43.3)		78 (38.8)	14 (38.9)	
TNM						
I+II	114 (55.1)	13 (43.3)	0.228	109 (54.2)	18 (50.0)	0.639
III+IV	93 (44.9)	17 (56.7)		92 (45.8)	18 (50.0)	
Chemotherapy						
No	112 (58.9)	17 (56.7)	0.813	118 (58.7)	21 (58.3)	0.967
Yes	85 (41.1)	13 (43.3)		83 (41.3)	15 (41.7)	

*Abbreviations*: *AFP* α-fetoprotein, *CEA* carcinoembryonic antigen, *TNM* tumor node metastasis, *ALB* albumin, *TBIL* total bilirubin, *ALT* alanine aminotransferase

### Relationship between Arg-1 and GPC-3 levels and overall survival

**Fig. 2:**
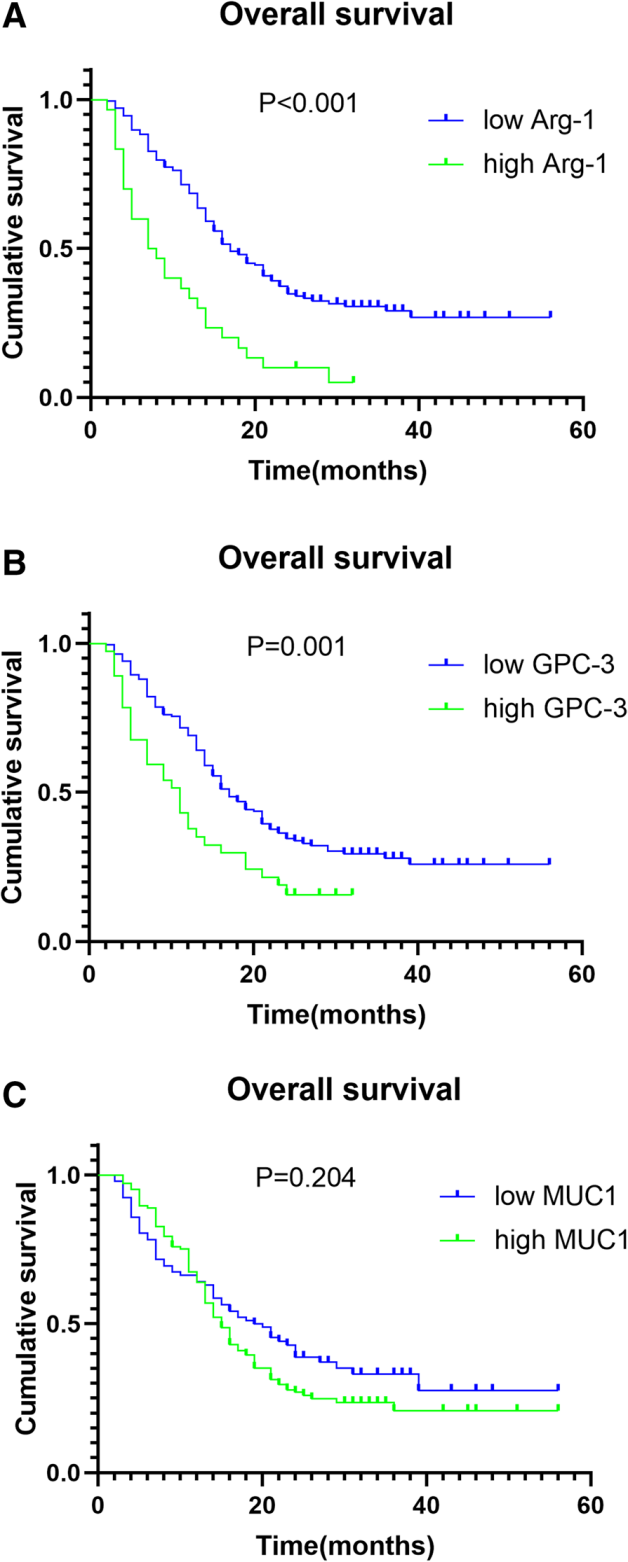
Kaplan-Meier survival curves showing the relationship between Arg-1, GPC-3, and MUC1 levels and overall survival (OS) in patients with intrahepatic cholangiocarcinoma. **A** Correlation of Arg-1 levels with OS. **B** Correlation of GPC-3 levels with OS. **C** Correlation of MUC1 levels with OS

The last follow-up time of this study was December 2020 and the median survival time was 16 months. Univariate analyses revealed that Arg-1 and GPC-3 levels, tumor size, tumor number, lymph node metastasis, vascular invasion, and tumor stage were associated with tumor growth. In contrast, MUC1 levels, age, gender, HBV infection, nerve invasion, and tumor differentiation were not significantly associated with ICC development. Arg-1 levels (hazard ratio [HR], 2.201; 95% confidence interval [CI], 1.438–3.371; *P* < 0.001) and GPC-3 levels (HR, 1.610; 95% CI, 1.061–2.442; *P* = 0.025) were independent prognostic variables associated with overall survival (OS) (Table 2). Kaplan-Meier analyses confirmed that high Arg-1 and GPC-3 expression levels were associated with short OS. In contrast, MUC1 levels were not significantly associated with OS (Fig. 2).

**Table 2:** Univariate and multivariate analysis of risk factors in relation to OS in ICC

Parameter	*N*	Univariate analysis		Multivariate analysis	
		HR (95% CI)	*P* value	HR (95% CI)	*P* value
**Gender**					
Female	112	1.145 (0.982‑1.336)	0.075		
Male	125				
**Age (years)**					
< 60	108	0.942 (0.694‑1.279)	0.695		
≥ 60	129				
**Arg-1**					
Low	207	2.620 (1.739‑3.948)	**< 0.001**	2.201 (1.438‑3.371)	**< 0.001**
High	30				
**GPC-3**					
Low	201	1.886 (1.267‑2.806)	**0.001**	1.610 (1.061‑2.442)	**0.025**
High	36				
**MUC1**					
Low	93	1.222 (0.889‑1.680)	0.204		
High	144				
**Tumor size (cm)**					
< 5	142	1.392 (1.024‑1.893)	**0.030**	1.115 (0.806‑1.544)	0.510
≥ 5	95				
**Tumor number**					
**Single**	167	1.379 (1.017‑1.890)	**0.046**	1.307 (0.941‑1.815)	0.110
**Multiple**	70				
**Lymph node metastasis**					
No	134	1.728 (1.273‑2.345)	**< 0.001**	1.321 (0.774‑2.255)	0.308
Yes	103				
**Vascular invasion**					
No	133	1.673 (1.233‑2.271)	**0.001**	1.187 (0.782‑1.801)	0.421
Yes	104				
**Nerve invasion**					
No	206	1.279 (0.829‑1.972)	0.252		
Yes	31				
**Differentiation**					
W+M	144	0.915 (0.671‑1.247)	0.563		
*P*	93				
**TNM**					
I+II	127	1.649 (1.214‑2.238)	**0.001**	1.162 (0.739‑1.825)	0.516
III+IV	110				
**AFP (ng/mL)**					
< 25	165	0.866 (0.623‑1.205)	0.381		
≥ 25	72				
**CEA (ng/mL)**					
< 5	134	1.172 (0.863‑1.590)	0.297		
≥ 5	103				
**CA19-9 (IU/L)**					
< 37	141	1.201 (0.883‑1.635)	0.231		
≥ 37	96				
**TBIL (μmol/L)**					
< 17.1	92	0.908 (0.665‑1.240)	0.534		
≥ 17.1	145				
**ALB (g/L)**					
**< 35**	163	1.137 (0.823‑1.573)	0.424		
**≥ 35**	74				
**ALT (U/L)**					
**< 40**	96	1.098 (0.803‑1.502)	0.547		
**≥ 40**	141				
**Chemotherapy**					
No	139	0.895 (0.657‑1.220)	0.472		
Yes	98				

*Abbreviations*: *W* well differentiated, *M* moderately differentiated, *P* poorly differentiated, *ALB* albumin, *TBIL* total bilirubin, *ALT* alanine aminotransferase

### Relationship between Arg-1 and GPC-3 levels and disease-free survival

**Fig. 3:**
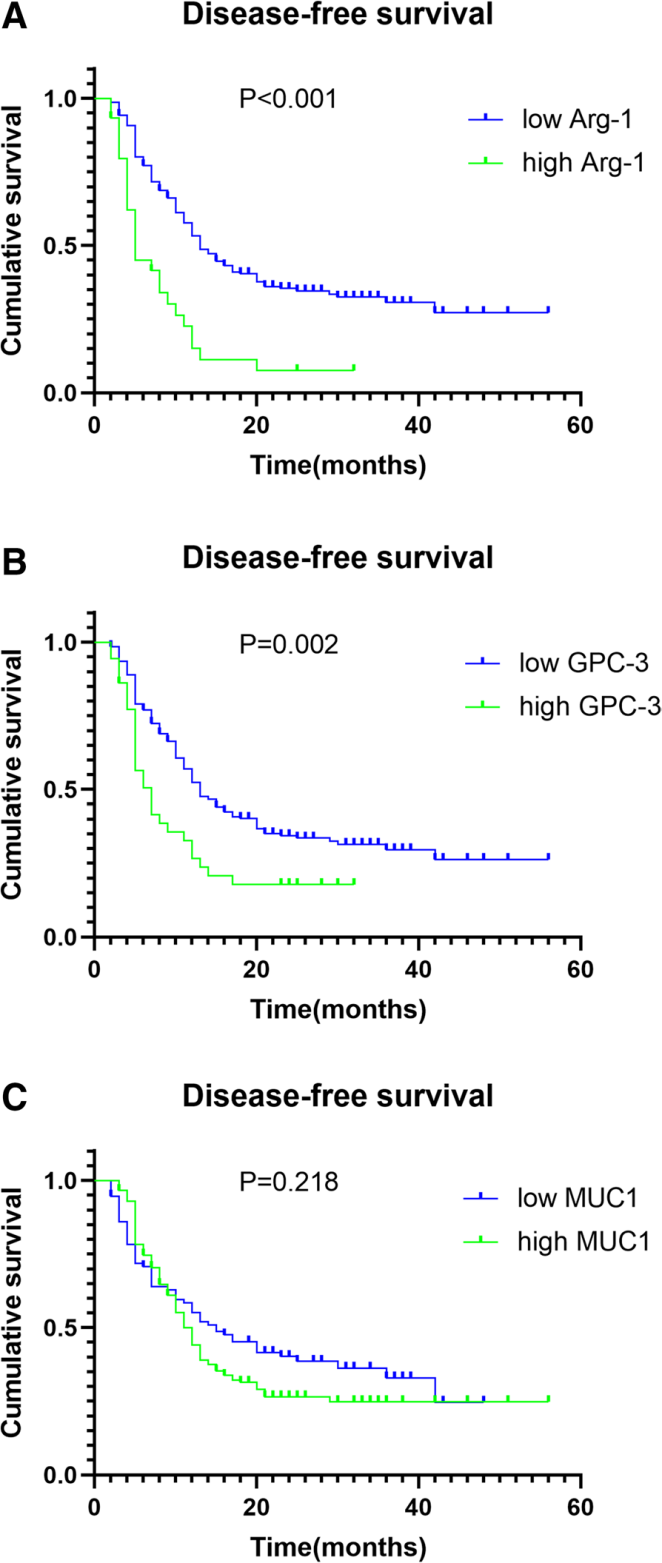
Kaplan-Meier survival curves showing the relationship between Arg-1, GPC-3, and mucin-1 levels and disease-free time survival (DFS) in patients with intrahepatic cholangiocarcinoma. **A** Correlation of Arg-1 levels with DFS. **B** Correlation of GPC-3 levels with DFS. **C** Correlation of MUC1 levels with DFS

High Arg-1 and GPC-3 levels, tumor grade, lymph node metastasis, vascular invasion, and TNM stage were risk factors associated with ICC progression. Arg-1 (HR, 2.258; 95% CI, 1.447–3.525; *P* = 0.001) and GPC-3 (HR, 1.548; 95% CI, 1.011–2.372; *P* = 0.045) levels were shown to be independent risk factors for DFS utilizing the Cox regression proportional hazards model (Table 3). Kaplan-Meier analyses indicated that high expression levels of Arg-1 and GPC-3 were associated with short disease-free survival (DFS). MUC1 levels were not significantly associated with DFS (Fig. 3).

**Table 3:** Univariate and multivariate analysis of risk factors in relation to DFS in ICC

Parameter	*N*	Univariate analysis		Multivariate analysis	
		HR (95% CI)	*P* value	HR (95% CI)	*P* value
**Gender**					
Female	112	1.155 (0.987‑1.351)	0.062		
Male	125				
**Age (years)**					
< 60	108	0.924 (0.677‑1.261)	0.606		
≥ 60	129				
**Arg-1**					
Low	207	2.665 (1.740‑4.082)	**< 0.001**	2.258 (1.447‑3.525)	**0.001**
High	30				
**GPC-3**					
Low	201	1.843 (1.222‑2.778)	**0.002**	1.548 (1.011‑2.372)	**0.045**
High	36				
**MUC-1**					
Low	93	1.218 (0.881‑1.684)	0.218		
High	144				
**Tumor size (cm)**					
< 5	142	1.362 (1.015‑1.864)	**0.045**	1.121 (0.808‑1.555)	0.493
≥ 5	95				
**Tumor number**					
Single	167	1.253 (0.899‑1.746)	0.167		
Multiple	70				
**Lymph node metastasis**					
No	134	1.890 (1.383‑2.583)	**< 0.001**	1.474 (0.852‑2.550)	0.165
Yes	103				
**Vascular invasion**					
No	133	1.780 (1.303‑2.432)	**< 0.001**	1.222 (0.798‑1.872)	0.356
Yes	104				
**Nerve invasion**					
No	206	1.212 (0.772‑1.902)	0.387		
Yes	31				
**Differentiation**					
W+M	144	0.845 (0.617‑1.158)	0.278		
*P*	93				
**TNM**					
I+II	127	1.694 (1.240‑2.314)	**0.001**	1.146 (0.722‑1.820)	0.564
III+IV	110				
**AFP (ng/mL)**					
< 25	165	0.874 (0.623‑1.226)	0.418		
≥ 25	72				
**CEA (ng/mL)**					
< 5	134	1.184 (0.867‑1.617)	0.271		
≥ 5	103				
**CA19-9 (IU/L)**					
< 37	141	1.171 (0.854‑1.604)	0.310		
≥ 37	96				
**TBIL (μmol/L)**					
< 17.1	92	0.892 (0.650‑1.224)	0.465		
≥ 17.1	145				
**ALB (g/L)**					
< 35	163	1.202 (0.865‑1.669)	0.256		
≥ 35	74				
**ALT (U/L)**					
< 40	96	1.022 (0.744‑1.402)	0.891		
≥ 40	141				
**Chemotherapy**					
No	139	0.888 (0.647‑1.219)	0.446		
Yes	98

## Discussion

ICC etiology is complex and has not yet been elucidated. As the second largest primary malignant tumor of liver, many studies have explored the prognostic factors of ICC, including inflammatory biomarkers, surgical risk scores, and pathological classification of ICC [15–18]. These previous studies provided some help for improving the prognosis of ICC, but its effectiveness still needs to be further explored. Arg-1 and GPC-3 have been extensively used to differentiate hepatocellular carcinoma cells from cholangiocarcinoma and metastatic tumor cells in the liver because of their sensitivity [19, 20]. However, their prognostic value in cholangiocarcinoma remains unclear.

Overexpression of Arg-1 has been linked to poor prognosis in certain cancers, including colorectal cancer and breast cancer [21, 22]. Tao et al. [23] found that high Arg-1 expression levels in hepatocellular carcinoma were associated with poor tumor differentiation and poor prognosis. Here, we found that high Arg-1 expression levels were strongly associated with shorter OS and DFS in patients with ICC, suggesting that Arg-1 may promote ICC development and progression. Arg-1 was found to play a key role in the urea cycle and participate in amino acid metabolism. Recent studies have shown that it may be involved in the occurrence and progression of tumors [24]. The high expression of Arg-1 could consume l-arginine in the tumor microenvironment, seriously inhibited the function of T cells and promoted the occurrence of tumor escape. The deficiency of l-arginine results in downregulated expression of the T cell receptor (TCR) light chain, which is the main signal transduction component of TCR. The downregulated expression of TCR light chain reduced the reactivity of T cells to antigens or mitogens, resulting in the reduction of tumor specific immune response, so as to decrease tumor clearance and promote tumor progression [25]. Therefore, Arg-1 may affect the prognosis of ICC patients through the regulation of tumor immune microenvironment, which plays an important role in the development of ICC [26, 27]. Other studies have shown that l-ornithine produced by Arg-1 could be further metabolized into polyamines, which were important components in cell differentiation and proliferation as well as promoting tumor growth [28]. We will continue to conduct more in-depth research on the mechanism.

Glypican belongs to the heparin proteoglycan family and is composed of a core protein and two heparin sulfate (HS) glycosaminoglycan chains [29]. GPC-3 was first identified in mouse epithelial cell lines by Filmus et al. in 1988 [30]. GPC-3 is highly expressed in many tumor tissues, and it is expressed in low levels in normal human tissues. Many studies have shown that GPC-3 promotes hepatocellular carcinoma cell proliferation by activating the classical Wnt signaling pathway [31, 32]. Wang et al. [33] reported that GPC-3 directly upregulated β-catenin to promote the proliferation and growth of lung squamous cell carcinoma. GPC-3 has also been reported to enhance the proliferation of nephroblastoma, hepatoblastoma, and melanoma cells [34, 35]. In our study, we found the similar results that high GPC-3 expression levels were associated with tumor size and poor OS and DFS. The finding suggests that GPC-3 may promote the growth and metastasis of ICC cells by activating the Wnt signaling pathway. However, the specific mechanisms remain to be characterized. However, Stigliano et al. [36] showed that GPC-3 inhibited the invasion and metastasis of breast cancer cells by reexpression in breast cancer LM3 cells and activating the non-canonical Wnt signaling pathway. Differences in cell types may have contributed to these contradicting findings. In the present study, we found that tumor size, tumor number, TNM staging, lymph node metastasis, and vascular invasion were risk factors for the prognosis of ICC, but not independent risk factors. Compared to our findings, Geers et al. reported that locoregional LNM was the only significant independent prognostic factor to determine both OS and DFS in perihilar cholangiocarcinoma [37]. The reason might be that there were many differences in the study population.

In addition, we investigated the prognostic value of mucin-1 expression levels in ICC. MUC1 is the earliest known protein in the mucin family. Under normal conditions, MUC1 is expressed in glandular epithelial cells in many tissues and organs [38]. Many studies have shown that MUC1 regulates tumor cell proliferation and metastasis [39–41]. Beatson et al. [42] showed that MUC1 promoted immune escape in cholangiocarcinoma tumor cells by upregulating PD-L1 and metastasis-associated proteins. MUC1 was associated with poor prognosis in ICC. In the present study, we found that MUC1 levels had no prognostic value in ICC. We believe that the following reasons may cause this difference. First, the small sample size may affect the effectiveness of the analysis to a certain extent. Second, the difference of human race may also lead to this different conclusion. Most liver tumors in Western population are alcohol-related or hepatitis C virus-related liver cancer, while most of the Eastern population are hepatitis B virus related. The biological characteristics and behavior of tumors caused by different causes may be different. Our study is based on Asian population. Hence, whether the expression status and action pathway of MUC1 in Western patients are the same as Asian ones remains to be studied. Finally, there are differences in immunohistochemical staining, such as the types of reagents used and scoring methods, it may lead to the heterogeneity of results.

There are certain limitations in our research. First, this study was a single-center retrospective study, potentially causing the introduction of selection bias. Therefore, multicenter prospective studies are required to confirm our findings. Second, the mechanisms by which Arg-1 and GPC-3 promote tumor growth, metastasis, and recurrence in ICC patients were not evaluated in this study. In vitro and in vivo studies are required to explore the role of Arg-1 and GPC-3 in ICC progression. Third, due to the low expression of Arg-1 and GPC-3 in cholangiocarcinoma tissues, the sample size was small. Large cohort studies are warranted to confirm the clinical significance of Arg-1 and GPC-3 in patients with ICC.

## Conclusions

In summary, our findings suggest that Arg-1 and GPC-3 can be used as independent markers to evaluate the prognosis of patients undergoing ICC surgical resection. Although Arg-1 and GPC-3 may be associated with ICC progression and metastasis, the underlying mechanisms remain unclear. Future studies are needed to explore the role of Arg-1 and GPC-3 in ICC progression and evaluate their potential use as therapeutic targets to treat ICC.

## Supplementary Information

**Additional file 1: Fig. 2** The adjusted survival curves.

## Data Availability

Not applicable

## Abbreviations

Arg-1: Arginase-1
GPC-3: Glypican-3
ICC: Intrahepatic cholangiocarcinoma
MUC-1: Mucin-1
IHC: Immunohistochemistry
OS: Overall survival
DFS: Disease-free survival
AFP: α-Fetoprotein
CEA: Carcinoembryonic antigen
TNM: Tumor node metastasis
