# Supplementary figures and images for: The prognostic value of arginase-1 and glypican-3 expression levels in patients after surgical intrahepatic cholangiocarcinoma resection
Source: World J Surg Oncol. 2021 Oct 29;19:316. doi: 10.1186/s12957-021-02426-9 (PMC8556943; doi:10.1186/s12957-021-02426-9)

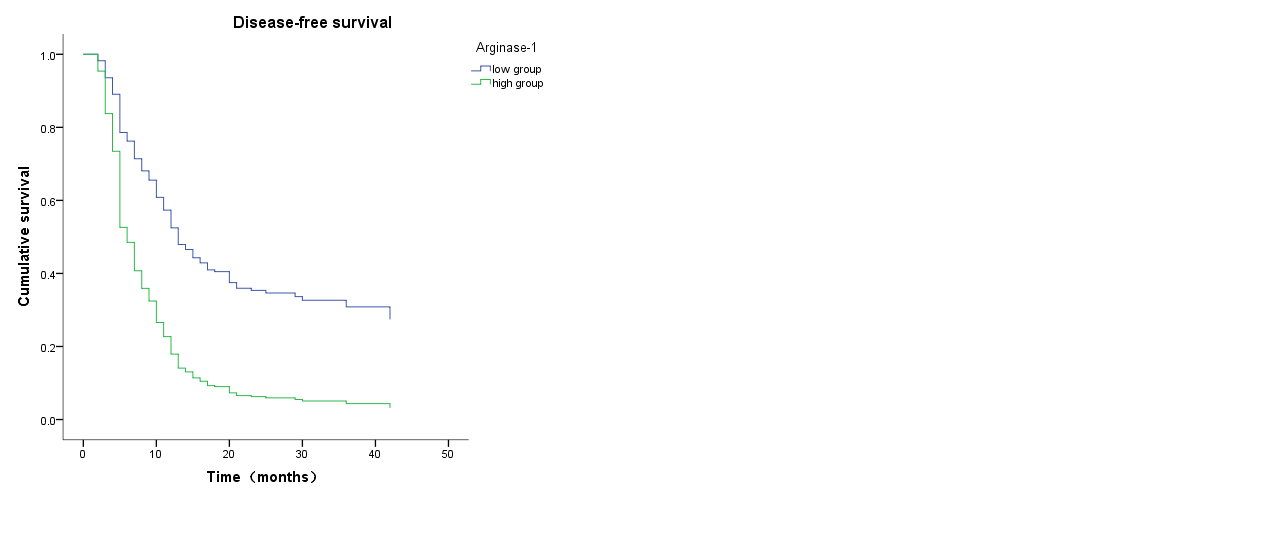

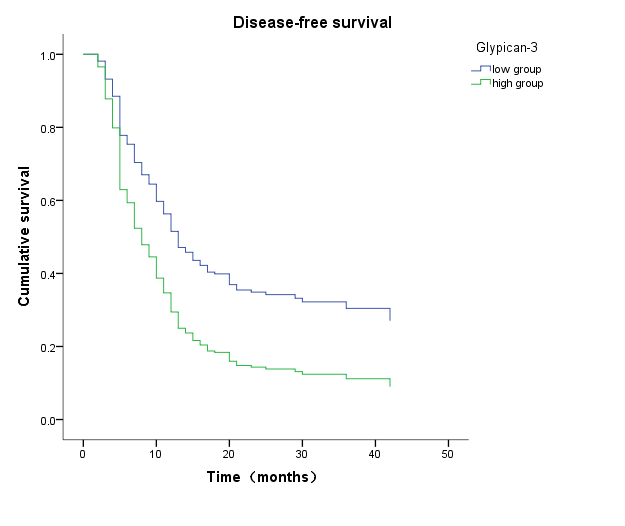


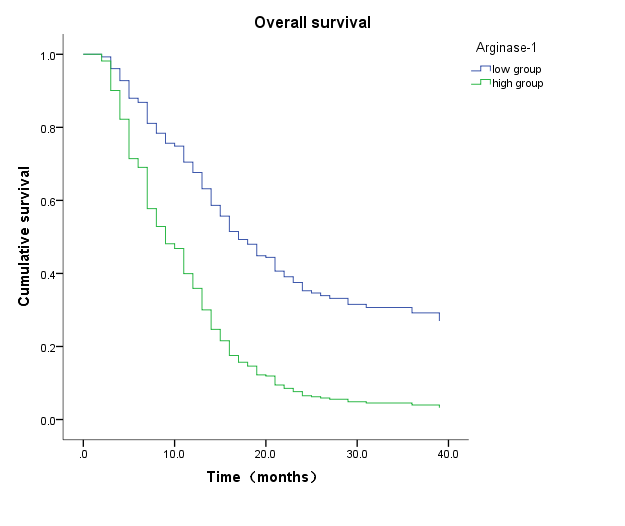

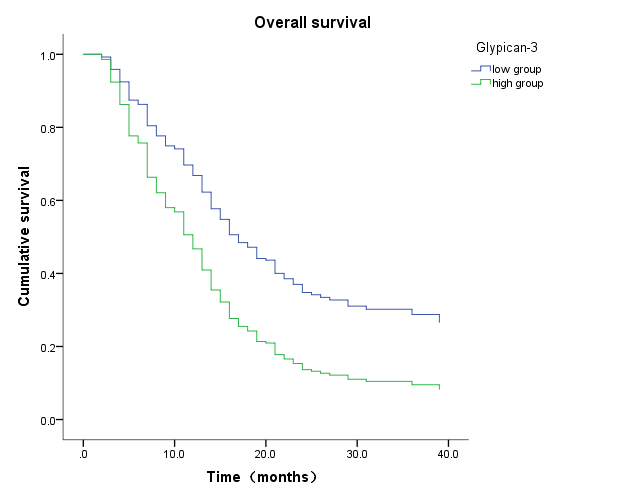


Fig. 2 The adjusted survival curves.

Supplement: Supplementary file 1 — Additional file 1: Fig. 2 The adjusted survival curves. [file 12957_2021_2426_MOESM1_ESM.docx]
